# Antibody Targeting of “Steady-State” Dendritic Cells Induces Tolerance Mediated by Regulatory T Cells

**DOI:** 10.3389/fimmu.2016.00063

**Published:** 2016-02-23

**Authors:** Karsten Mahnke, Sabine Ring, Alexander H. Enk

**Affiliations:** ^1^University Hospital Heidelberg, University of Heidelberg, Heidelberg, Germany

**Keywords:** dendritic cells, regulatory T cells, antibody targeting, DEC205, tolerance

## Abstract

Dendritic cells (DCs) are often defined as pivotal inducers of immunity, but these proinflammatory properties only develop after stimulation or *ex vivo* manipulation of DCs. Under non-inflammatory conditions *in vivo*, DCs are embedded into a tissue environment and encounter a plethora of self-antigens derived from apoptotic material. This material is transported to secondary lymphoid organs. As DCs maintain their non-activated phenotype in a sterile tissue environment, interaction with T cells will induce rather regulatory T cells than effector T cells. Thus, DCs are not only inducers of immunity but are also critical for maintenance of peripheral tolerance. Therapeutically, intervention for the induction of long-lasting tolerance in several autoimmune conditions may therefore be possible by manipulating DC activation and/or targeting of DCs in their “natural” tissue environment.

## Introduction

Mature dendritic cells (DCs) are perfectly equipped with MHC class II–peptide complexes and T cell interacting molecules (immune stimulatory and immune regulatory ones) to regulate T cell activation and differentiation in secondary lymphoid organs. The DCs act as sentinels in the periphery of the body where they take up antigens and sample the cellular environment. From here, antigens are transported into secondary lymphoid organs, and the subsequent interaction with T cells is critical for induction of immunity as well as for induction of tolerance. Initial investigations established DCs as pivotal inducers of immunity but with more molecules discovered on the surface of DCs and refined immunological methods, a role in the induction of regulatory T cells (Treg) became clear (1).

The initial experiments on DC functions involved the isolation of DCs from tissues and the *ex vivo* generation from either bone marrow cells (mouse) or monocytes (human). But these *in vitro* methods inherently stimulate and activate the developing DCs and when analyzed in T cell stimulation assays, activating capacity was recorded for most DC types.

In contrast to *in vitro* cultures, *in vivo* DCs are embedded in a tissue and additional activating stimuli, i.e., TLR ligands and/or trauma have to be applied to DCs *in situ* in order to mature them and to stimulate their proinflammatory functions. This maturation process is fast (approx. 6 h) and effective (2). It induces migration to secondary lymphoid organs and surface expression of MHC class II and T cell costimulatory molecules (3). Therefore, approaches to isolate naïve, non-activated DC directly from tissues is nearly impossible, as manipulation provides enough stimuli to trigger maturation.

## *In Vivo* Targeting of Antigens to Immature DCs Induce Treg

**Figure 1:**
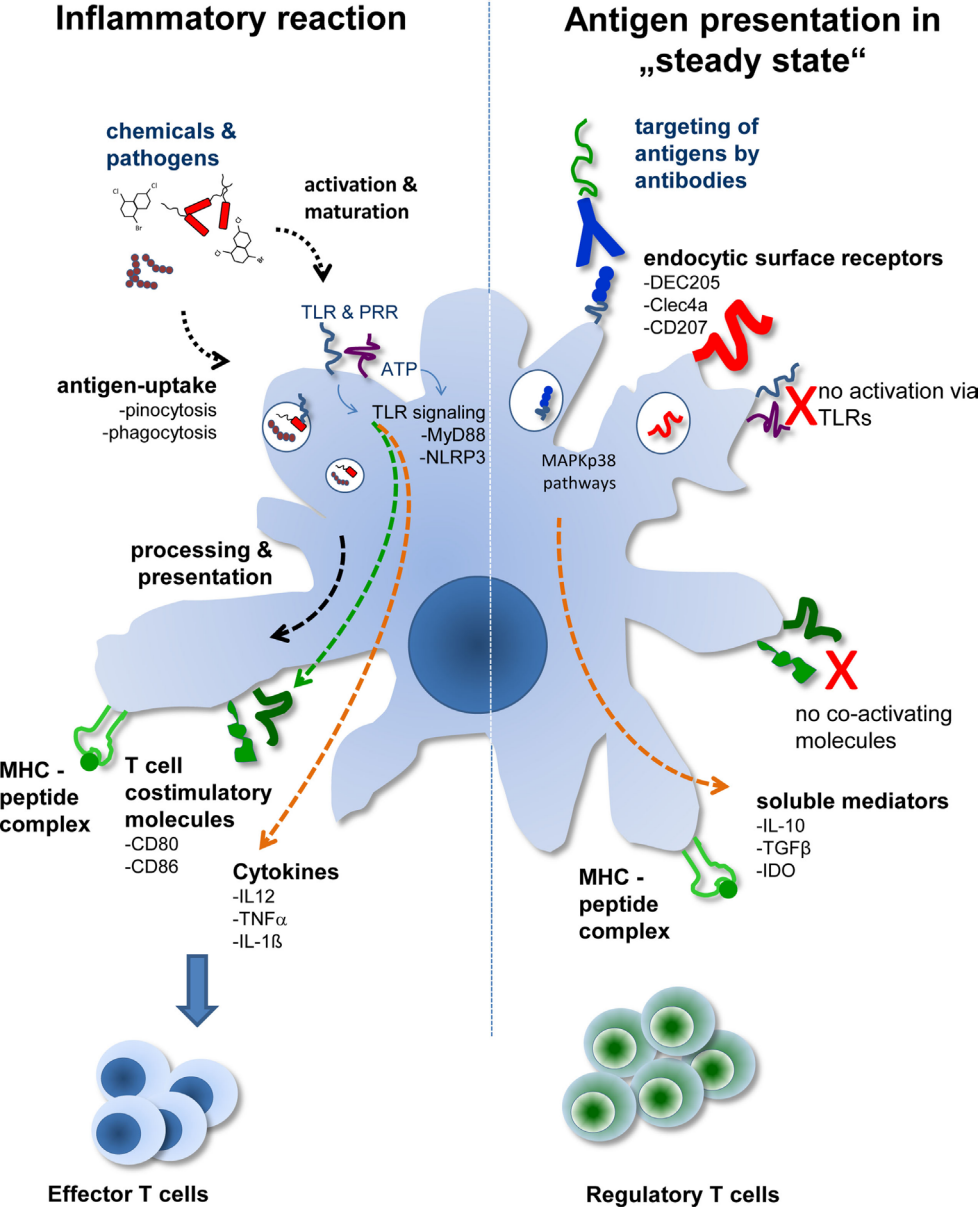
**The two different faces of DCs**. Depending on the activation status and/or antigen delivery immunity or Treg induction ensues. During tissue disruption or infection, antigens are delivered to DCs together with activating signals. This leads to activation of DCs marked by the production of proinflammatory cytokines and expression of T cell costimulatory molecules. During sterile, i.e., steady-state conditions, transportation of antigens to DCs *in situ* by DC-specific antibodies leads to uptake and antigen presentation by DCs without activating them. In the absence of T cell costimulatory molecules and further support by soluble mediators, such as TGF-β or IL-10, Treg are generated by “steady-state” DCs.

A fundamentally different approach from isolating DCs and pulsing them with respective antigens is the technique to load DCs *in vivo* by antibody targeting. This approach, so far mostly tested in animal models, mimics the *in situ* situation better. Here, DCs remain embedded into a tissue environment, and no further stimuli, such as trauma and/or infection, are present. In this sterile environment, the antigen is delivered by binding to a DC-specific antigen-uptake receptor. One of the first receptors used in this approach is the DEC205 receptor (4, 5). DEC205 is a lectin-like receptor and is closely related to the macrophage-mannose receptor. DEC205 recycles through late endosomal MHC class II^+^ compartment and effectively promotes antigen presentation by DCs (6). Therefore, techniques were developed to couple antigens to DEC205-specific antibodies and to inject these conjugates into animals (Figure 1).

In initial experiments model antigens, such as hen egg lysozyme or ovalbumin, which are frequently used in immunology, were coupled to anti-DEC205 antibodies. In these experiments it was proven that anti-DEC coupled antigens (anti-DEC:antigen) targeted DCs *in situ* and that the antigens were indeed taken up by the DCs (7–9). This process was highly specific as uptake of anti-DEC:antigen conjugates by Fc receptor expressing cells, such as macrophages, B cells, or granulocytes, was negligible. When anti-DEC:antigen conjugates were injected into animals without further adjuvant the DC phenotype remained non-activated and T cell costimulatory molecules, such as B7.1 and B7.2, showed expression levels comparable to those obtained in DCs from control mice. However, the *steady-state* DCs were not immunologically inactive, because analysis of antigen-specific T cell populations in injected mice revealed increased numbers of Treg. These T cells expressed the *bona fide* Treg markers CD4, CD25, IL-10, and FoxP3, and were able to suppress proliferation of CD4^+^ T cells *in vivo* (8, 10). Thus, antigen presentation by *steady-state* DCs *in vivo* induces Treg, which offers the possibility to provide a tool for tolerogenic cell therapy in humans.

## Antibody Targeting to DC as Therapeutic Tool in Autoimmune Diseases

To assess the possibilities of DEC targeting in therapeutic settings, self-antigens were coupled to anti-DEC205 antibodies and conjugates were tested in respective animal models. For instance, anti-DEC205 targeting and Treg induction thereof was tested in a transgenic mouse model for diabetes. In this model, transgenic mice express hemagglutinin (HA) under control of the RIP promoter in pancreatic islets and develop insulinitis (and finally diabetes) upon transfer of HA-specific T cells. When anti-DEC:HA conjugates were used in this model, insulinitis was prevented and diabetes did not develop (11).

Also in another autoimmune model, the experimental allergic encephalitis (EAE), which serves as model for multiple sclerosis, anti-DEC:antigen targeting lead to strong amelioration of the disease. Here, the naturally occurring autoantigen MOG was fused to DEC205-specific single chain fragment variables and mice were treated before and during EAE induction. It was found that IL-10 producing CD4/CD25/FoxP3^+^ Treg were induced, which prevented outbreak of EAE when animal were “tolerized” by anti-DEC:MOG before induction of the disease (12). Moreover, even when anti-DEC:MOG treatment was started after onset of EAE symptoms, a significant amelioration of the disease could be observed. Finally, targeting of the cartilage proteoglycan to DEC205^+^ DCs by means of antibody:antigen conjugates lead to prevention of autoimmune arthritis (13). In summary, these data establish DC targeting *via* DEC205 as an effective strategy to tolerize against autoantigens in order to protect against autoimmunity.

The DEC205 receptor is in particular suited for DC targeting, as it not only binds antigens to DCs, but also acts as a prototype antigen receptor that actively delivers potential ligands to MHC class II^+^ compartments. But recently also other surface molecules for DC targeting have been defined on several subsets of DCs. But most of them (e.g., DC-SIGN and CD40) are used for induction of immunity, as they may also possess DC-activating capacity (14). But for CLEC9A mediated targeting, both Treg-inducing and immune stimulatory effects have been described (15–17), and it remains unclear which co-factors and/or differentiation patterns of DCs are involved in regulating these opposite functions. However, some novel candidates have been shown to be able to induce Treg and to lead to tolerance. Among them are CD103, langerin (CD207) (18), triggering receptor on monocytic-like cells (Treml)-4, and Clec4A (19). All are expressed by DCs in varying degrees. However, the most effective DC populations for Treg induction were positive for CD103 and CD207. Interestingly, these molecules are predominantly expressed by tissue residing DCs (e.g., skin and/or gut), which are highly migratory and enter the secondary lymphoid organs on their normal trafficking routes (20).

## *Steady-State* DCs are Prone to Induce Treg-Mediated Tolerance

Irrespective of the specificity and the signaling capacity of the antibodies used for targeting, one common denominator for Treg induction by DCs after antibody targeting is their “steady state.” This became clear when the same antibody:antigen complexes, which were successfully used for tolerance induction, were injected together with DC-activating stimuli. For instance, when anti-DEC205 antibodies were coupled to tumor antigens, they were able to induce robust anti-tumor immunity when applied together with TLR ligands (21). Similar results were obtained with CLEC9A, which when applied under DC-activating conditions, induces strong humoral and cellular Th1 immunity (16, 17). Therefore, the activation status of the DCs may be critical for decision making on whether tolerance, or immunity ensues after DC–T cell interaction in secondary lymphoid organs.

These opposed functions of otherwise similar DC subsets (as defined by surface marker expression) led to a conceptual view that not particular subsets of DCs but rather non-activated or *steady-state* DCs are involved in generating Treg and tolerance. Similar to their function as immune stimulators, DCs act as sentinels in the periphery of the body (22). In the absence of inflammation or tissue injury, DCs are constantly exposed to self-proteins. These proteins are taken up and transported by DCs to secondary lymphoid organs. But in contrast to foreign and potentially dangerous antigens, the self-antigens are presented in a tolerogenic fashion to induce Treg and pave the way for further suppressive mechanisms. Altogether, this may be a crucial process to maintain peripheral tolerance (23).

In support of this notion, it has been reported that in peripheral tissues, DCs engulf self-antigens provided by microvesicles, exosomes (24, 25), and detritus of epithelial cells lining the gut and lung (26, 27). These antigens are transported and presented to T cells in the draining lymph nodes (28). In particular, apoptotic cells contain a collection of self-antigens and immunity against those antigens would be fatal. Therefore, further safeguards to prevent DC activation and immunity are in place. In case of apoptotic material, specialized receptors, such as CD36 and α_v_β_3_/α_v_β_5_ integrins expressed by DCs, detect components of apoptotic cells and induce intracellular signaling events to prevent maturation and cytokine production (29, 30). In more detailed studies, the molecules *Gas* and *Protein 6*, expressed by dying cells are recognized by MerTK, leading to signaling *via* the rather inhibitory STAT3 pathway and simultaneously inhibiting NFkB activation (31). In summary, apoptotic material will keep DCs non-activated and presentation of the “self-derived” apoptotic cargo will not activate effector T cells. Moreover, the *steady-state* DCs will induce CD4^+^CD25^+^ Treg, which in turn will now suppress potentially self-reactive T cells.

This conceptual view requires a constant flow of non-activated DCs from the periphery to secondary lymphoid organs for the upkeep of Treg activation. If this trafficking is compromised, autoimmunity will develop. That has been demonstrated in a transgenic mouse model, where skin derived DCs are constantly activated by genetic manipulation. Here, severe autoimmunity to skin tissue is induced (32). Moreover, in humans, the incidence of the autoimmune disease systemic lupus erythematosus (SLE) is strongly correlated to a chronically activated phenotype of DCs (33). On the whole, these data suggest that DCs constantly sample the repertoire of peripheral proteins and are an effective source of “self-peptide–MHC” complexes. By presenting these complexes in the steady state, DCs induce Treg and thus suppress potentially self-reactive T cells.

## DC Not Only Induce Treg but also Limit Their Numbers by Converting Them into Effectors

All these data clearly demonstrate that experimentally modified and/or specialized DC subsets exist that are able to induce Treg. However, Treg are not always stable in phenotype and function, as they can convert into proinflammatory cells and loose their suppressive potential. *In vivo*, during the development of type-1 and type-2 responses, Treg are exposed to a variety of cytokines and in this process, DCs play a role in driving the conversion of Treg into Th17-like cells either directly or indirectly. A rather indirect and cytokine mediated pathway involves IL-6, which can be secreted by activated DCs. This IL-6 secretion by DCs may contribute to Treg conversion as natural Treg develop into IL-17^+^, IFNγ^+^ proinflammatory cells after treatment with IL-6 *in vitro* (34, 35).

A direct mechanism by which DCs are involved in converting mouse CD25^+^Foxp3^+^ Treg into a proinflammatory cell type is demonstrated by dectin-1. This C-type lectin receptor is normally involved in fungal recognition. But in cocultures of Treg with DCs that have selectively been activated *via* dectin-1, IL-23 is produced. This leads to conversion of Treg into Th17-like effector T cells, characterized by the expression of ROR-γt and the production of IL-17 (36).

In addition to dectin-1, which is rather an antigen-uptake molecule and may therefore signal intracellular after uptake of antigens, surface molecules, such as the T-cell immunoglobalin mucin (TIM-1), may have direct outside-in signaling capacities to turn down Treg induction by DCs by means of Treg conversion. For TIM-1, it has been shown that a high-avidity/agonistic anti-Tim-1 antibody enhances the immunogenic function of DCs, leading to decreased suppressive function of Treg. At the same time, substantially increased conversion of Treg into proinflammatory IL-17^+^ T cells was observed (37).

Likewise, CD18 seems also to be involved in regulating DC-mediated Treg conversion into proinflammatory T cells (38). In a CD18-deficient mouse model, enhanced numbers of Th17 cells were observed resulting in a psoriasis-like phenotype. These data are backed by clinical observations in humans, showing that patients with LAD syndrome (leukocytes do not express functional CD18 in LAD patients) have both elevated levels of Th17 cell and psoriasiform skin disease. Further experiments revealed that this accelerated conversion of Treg into Th17 cells was due to an inadequate DC-Treg interaction *via* CD18 because blockade of CD18 interactions between DCs and Treg *in vitro* lead to rapid generation of IL-17^+^ “ex”-Treg.

However, not only the stability of Treg is influenced by DCs and their respective cytokine environment but also their induction is affected by the context of cytokines and/or pathogens. By broadening the concept that immature DCs are prone to induce Treg, it is conceivable that fully activated and mature DCs induce effectors and may even hamper Treg generation.

For instance, retinoic acid (RA) has been shown to promote conversion of naïve T cells into Treg cells, presumably by acting on DCs and augmenting TGF-β. Henceforth, studies have been undertaken to block RA receptors by an antagonist (RARi). RARi significantly suppressed TGF-β and IL-10, and enhanced IL-12 production by DC in a tumor model. This protective effect was associated with significant reduction in tumor-infiltrating FoxP3^+^ and IL-10^+^ Treg cells, and a corresponding increase in tumor-infiltrating CD4^+^ and CD8^+^ T cells that secreted IFN-γ (39).

Also, vigorously enhanced expression of T-cell costimulatory molecules by DCs provides a means by which Treg activation is suppressed. In the study of Pen et al. (40), DCs were transfected with constitutively active TLR4, CD40 ligand, and the costimulatory molecule CD70. These DCs could partly alleviate Treg inhibition of CD8^+^ T cells, which was further accompanied by a decrease in CD27 and CD25 expression on Treg. Moreover, an increase in the expression of T-bet and secretion of IFN-γ, tumor necrosis factor (TNF)-α, and IL-10 was recorded, suggesting a shift of the Treg phenotype toward a Th1 phenotype.

Finally, even without molecular transfection approaches, CpG-ODN-stimulated DCs exhibited pivotal proinflammatory functions, as in a *Leishmania donovani* model intracellular parasitic growth was abolished by these CpG-stimulated DCs (41). Alongside, it was observed that DC vaccination resulted in significant decreased Treg numbers. Moreover, the remaining Treg were partly defective in TGFβ secretion and were affected in IP-10 signaling.

Although the exact molecular mechanism are not clear yet, evidence exists that p38 expression in DCs is crucial for regulating their Treg-activating and/or Treg-converting functions (42, 43). Therefore, small molecules tampering with p38 pathways activation in DCs may be useful to shift the Treg–Th17 balance *in vivo*.

Vice versa, in Treg expression of SOCS-1 (44), TRAF6 (45) and other transcriptions factors, such as Runx1, CNS2, and Cbfβ (46) is important for maintenance of their suppressive phenotype, because absence of these factors in murine knock out models resulted in increased conversion of Treg into Th17-like cells. Thus, future investigations have to address as to how DC subsets and/or surface molecules thereof interfere and trigger those signaling pathways in Treg.

In aggregate, these findings indicate that not only the generation of Treg but also their stability of phenotype is regulated by DCs. DC-derived cytokines, such as IL-6 and IL-23, as well as surface molecules are important to regulate Treg conversion into Th17 cells and are able to shift the balance between effector and Treg toward an enhanced immune response.

## *Ex Vivo* Manipulation of DCs for the Induction of Treg

### Role of Cytokines

In line with the concept that immature DCs induce Treg and tolerance, attempts can be made to stabilize the immature DC phenotype by manipulation of the cytokine environment. One important cytokine in this context is interleukin (IL)-10. After exposure of DCs to IL-10 *in vitro* culture systems, the cells display reduced surface expression of MHC class I and II molecules and reduced expression of T cell costimulatory molecules of the B7 family as compared to their untreated counterparts. But not only surface molecules change, also the release of proinflammatory cytokines, i.e., IL-1β, IL-6, and TNFα and most markedly IL-12, is abolished after IL-10 treatment (47). However, all of these effects could only be induced in immature DCs. In contrast, mature DCs are insensitive to IL-10 and display a stable phenotype in the presence of IL-10. Therefore, for therapeutically use, IL-10 has to be added to *in vitro* culture systems very early on and/or *in vivo* elevated IL-10 levels have to be present once pre-DC are present in the respective tissues. The proof that those immature DCs do not only possess weak proinflammatory properties but also actively induce T cells with regulatory properties comes from observations in human melanoma. For example, IL-10 producing metastases are able to convert DCs into tolerogenic DC, which induce anergy in melanoma or alloantigen-specific CD4^+^ and CD8^+^ T cells (48, 49). But these anergic T cells were not just inactive, instead they acted as “bystander” suppressors that actively curbed anti-melanoma T cell activation (50). Moreover, in mice overexpressing IL-10, DCs displayed a remarkably immature phenotype (51) and the appearance of the immature DCs was accompanied by substantially increased numbers of Treg in the spleens of these mice. This suggests that IL-10 plays an important role in rendering DCs not merely immature but also modifies their ability to induce Treg *in vivo*. To exploit the effects of IL-10 on DCs for Treg induction, IL-10 modulated DCs were injected in the murine model for EAE and in a GVHD model. In both instances, substantial amelioration of the disease and immune suppression by regulatory active T cells was observed (49, 52). Likewise, in a mouse model of asthma, the injection of IL-10-treated DCs lead to increased differentiation of *bona fide* Treg from effector T cells, which were able to ameliorate asthmatic incidence in house dust mite allergic mice (53, 54). Thus, these data establish IL-10 as a critical agent for induction of a Treg-inducing DC phenotype. But moreover, IL-10 may also facilitate the cross talk between Treg and DCs, since it acts simultaneously on both cell types. For example, Misra et al. (55) have shown that DCs cocultured with Treg remain in an immature state as judged by surface marker expression. These “Treg-exposed” DCs were inferior in induction of T cell proliferation and produced significant amounts of IL-10, which (i) maintains Treg function in a paracrine fashion and (ii) keeps DCs immature *via* an autocrine loop.

### Role of Vitamin D3

Beyond the IL-10 effects, also non-cytokine immune modulators are active in keeping DCs functional as “Treg-inducers.” For instance, vitamin D [VitD3: 1,25(OH)_2_D3] treated DCs display a rather immature phenotype as they express low MHC class II, low amounts of T cell costimulatory molecules, and high production of IL-10 (56). This clearly tolerogenic phenotype of DC exert their immune regulatory functions by different means. First, it has been shown that VitD3 DCs are inactive in priming CD4^+^ and CD8^+^ T cells. Second, they are inducers of apoptosis in effector T cells and thirdly they are able to induce allo- and autoantigen-specific Treg from naïve CD4^+^ T cells, which were able to block diabetes in a non-obese diabetes animal model (57–59).

In case of VitD3 DCs, TNF-α plays a critical role as co-inducer of Treg. Although TNFα has a long standing track record for being proinflammatory, it has been shown to act tolerogenic on DCs. First observations by Menges et al. showed that TNFα matured DC are able to ameliorate multiple sclerosis symptoms in a murine EAE model (60). Further observations with human cells broadened these findings, by showing that VitD3 increased the amounts of membrane-bound TNFα in DCs and that in particular, this membrane-bound TNFα is critical for the induction of Treg (53). In addition to VitD3 alone, DCs have also been treated in combination with Dexamethason. These combinatorially treated DCs exhibited a more stable tolerogenic phenotype *in vivo* and were more potent in inducing Treg in a murine colitis model (61).

## Conclusion

In summary, these data show that agents, which actively block DC maturation, augment the ability of the DCs to induce Treg. Therefore, the “default” function of immature DCs is to maintain sufficient numbers of Treg in the periphery of the body. Prevention of DC maturation as well as antigen loading of otherwise untouched *steady-state* DCs *in vivo* may therefore be one future therapeutic approach to induce long-lasting tolerance in different autoimmune diseases.

## Author Contributions

KM and AHE wrote the paper. SR prepared figures and helped writing the paper.

## Conflict of Interest Statement

The authors declare that the research was conducted in the absence of any commercial or financial relationships that could be construed as a potential conflict of interest.
